# Ionic fragmentation products of benzonitrile as important intermediates in the growth of polycyclic aromatic hydrocarbons†

**DOI:** 10.1039/d3cp05574d

**Published:** 2024-02-06

**Authors:** Daniël B. Rap, Johanna G. M. Schrauwen, Britta Redlich, Sandra Brünken

**Affiliations:** a Radboud University, FELIX Laboratory, Institute for Molecules and Materials Toernooiveld 7 6525 ED Nijmegen The Netherlands sandra.bruenken@ru.nl

## Abstract

In various astronomical environments such as the interstellar medium or (exo)planetary atmospheres, an interplay of bottom-up growth and top-down destruction processes of (polycyclic) aromatic hydrocarbons (PAHs) takes place. To get more insight into the interplay of both processes, we disentangle the fragmentation and formation processes that take place upon dissociative ionization of benzonitrile. We build on previous spectroscopic detections of the ionic fragmentation products of benzonitrile and use these as reactants for low-temperature bottom-up ion–molecule reactions with acetylene. By combining kinetics and infrared action spectroscopy, we reveal exothermic pathways to various (polycyclic) aromatic molecules, including the pentalene and phenylacetylene radical cations. We determine the reaction rate coefficients and unambiguously assign the structures of the reaction products. The data is supplemented by potential energy surface calculations and the analysis of non-covalent interactions. This study shows the unexpected formation of a linked four- and six-membered ring structure (phenylcyclobutadiene radical cation) with molecular formula C_10_H_8_˙^+^, and not the commonly observed isomer naphthalene˙^+^. All observed reactions proceed *via* radiative association processes and are relevant for the chemistry in (cold) astrochemical environments.

## Abstract 2

In various astronomical environments, an interplay of bottom-up growth and top-down destruction processes takes place. To unravel this, ionic fragments of benzonitrile are used as reactants for low-temperature ion-molecule reactions with acetylene.

## Graphical abstract

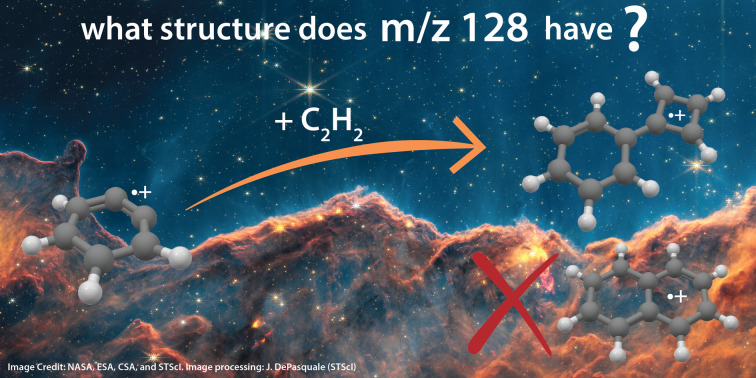

## Introduction

The chemistry in an environment can be dictated by various physical parameters such as temperature, pressure and the intrinsic properties of the molecules involved. The abundance of a particular species in a chemical system can be modeled and understood by its rates of formation, but also its rates of destruction. This interplay between formation and destruction also governs the chemistry of various astronomical environments such as the interstellar medium (ISM) and (exo)planetary atmospheres, and is often cited as bottom-up formation and top-down fragmentation processes,^1–3^ where bottom-up describes the formation of larger species from small building blocks and top-down involves the fragmentation of large species to smaller products.

The chemistry within these astronomical environments is not yet fully understood. This is especially clear from the observationally derived molecular abundances of the aromatic molecule benzonitrile^4^ and the recently detected polycyclic aromatic hydrocarbons (PAHs) 1- and 2-cyano-naphthalene^5^ in the cold molecular cloud TMC-1 that differ by orders of magnitude compared to current astrochemical models. This is likely due to the insufficient physicochemical knowledge on the (polycyclic) aromatic molecules involved, *e.g.*, unknown formation pathways, destruction processes and their accompanying branching ratios and efficiencies/rate coefficients. Both theoretical and experimental approaches are required to obtain these properties in order to establish astrochemical models that give a good understanding of the chemistry in those environments.^6^

There exist already several studies where formation pathways of (nitrogen-containing) PAHs (NPAHs) have been studied in detail,^7–16^ providing structural information of the products, branching ratios and rate coefficients that help to improve the astrochemical models. Other studies have been looking into the destruction processes of astronomical relevant species to understand their stability and fragmentation processes.^17–28^ Fragmentation processes are shown to play a role for the astronomically detected Buckminsterfullerenes C_60_, C_70_ and C_60_^+^ that are thought to be formed *via* top-down chemistry from large PAHs.^3,29,30^

In a chemical environment like the interstellar medium the fragmentation and formation reactions are strongly intertwined. The destruction of molecules may lead to dominant fragments that in turn can be involved in formation processes towards other, larger molecules. Top-down chemistry has been observed in the Horsehead photo-dissociation region (PDR). A key intermediate in carbon chemistry, l-C_3_H^+^, was observed to be more abundant in the PDR layers than predicted by astrochemical models, and to be spatially linked with PAHs rather than small neutral hydrocarbon molecules.^2^ This is consistent with additional top-down chemistry by UV photodissociation of PAHs that increases the abundance of this important intermediate species which in turn can be involved in gas-phase bottom-up chemistry in those sources.^2,31^ Eventually, this may lead to a family of stable molecules that resist destruction processes and/or chemical reactions. Even within dense clouds, where UV photons cannot penetrate, cosmic rays provide energy and can initiate ion–molecule reactions,^32^ enhance chemical reactions^33^ and trigger nonequilibrium chemistry in interstellar ices.^34^

Examples of experimental approaches that studied the linked formation and destruction chemistry involve for example electrical discharge^35–39^ and pyrolysis sources (microreactors),^40–43^ where fragmentation and recombination processes dominate the chemistry. However, detailed insight into which molecular structures play a dominant role is difficult to gain as one may predominantly see the thermodynamically stable products upon probing. By separating the dissociation and the recombination processes, individual steps can be studied in more detail. In a previous study, we have investigated the fragmentation processes of benzonitrile in detail using a combination of mass-selective infrared action spectroscopy and theoretical methods.^28^ This led to the spectroscopic identification of the cationic fragment structures from the major HCN/HNC loss channel upon dissociative ionization of benzonitrile. The cationic fragment structures with molecular formula C_6_H_4_˙^+^ were identified as two benzyne˙^+^ structures, *ortho*(*o*)- and bicyclic *meta*(*m*)-benzyne˙^+^, which are important players in PAH formation chemistry.^11,41,44^

**Fig. 1:**
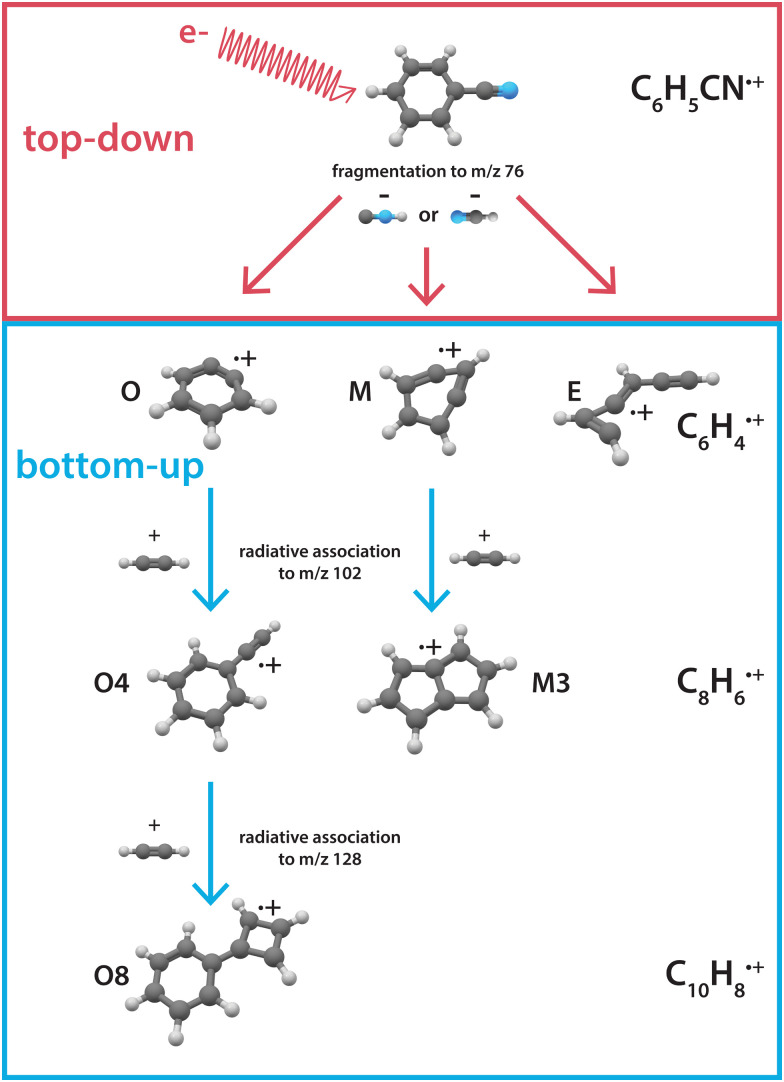
Illustration showing the interplay between top-down processes of benzonitrile^28^ and subsequent bottom-up formation reactions towards polycyclic aromatic hydrocarbons as discussed here. The labels correspond to the structures displayed in the PES's.

Here, we investigate the recombination chemistry of these ionic benzyne fragments, now regarded as the reactants, with neutral acetylene as a building block. We use electron impact (EI) dissociative ionization to generate the reactive ionic fragments *via* the HCN/HNC fragmentation channel of benzonitrile. These reactive precursors are mass-selected, trapped in a temperature-controlled 22-pole ion trap, and subsequently reacted with acetylene gas at a low temperature of 150 K.^45^ The formation of larger molecules is investigated using a combination of mass-spectrometric kinetic studies to obtain reaction rate coefficients and infrared action spectroscopy to experimentally elucidate the structures of the intermediates and products. The observed reaction processes are further investigated using quantum chemical methods. Fig. 1 shows an illustration of the top-down fragmentation processes of benzonitrile upon dissociative ionization through HCN/HNC loss, and the sequential bottom-up formation processes of this study.

## Methods

### Kinetic measurements

**Fig. 2:**
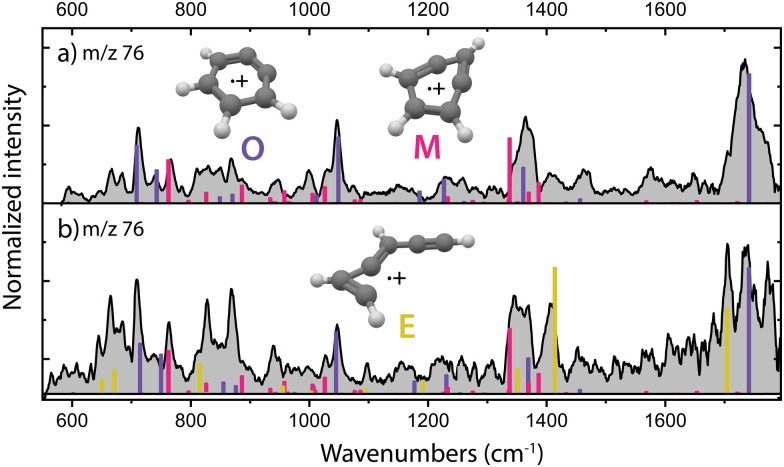
Experimental infrared spectra of the HCN/HNC fragmentation products (C_6_H_4_˙^+^, *m*/*z* 76, grey) of benzonitrile (C_6_H_5_CN˙^+^) formed in (a) the direct EI ionization source and (b) an EI ion storage source. Both mixtures of isomers have been used here as reactants for ion–molecule reactions with acetylene to study the formation of PAHs. Calculated vibrational modes of the assigned molecular structures *o*-benzyne˙^+^ (O, purple, +52.2 kJ mol^−1^), bicyclic *m*-benzyne˙^+^ (M, pink, +11.5 kJ mol^−1^) and ethynyl–methylene–cyclopropene˙^+^ (E, yellow, +15.3 kJ mol^−1^) are shown. The calculations of *o*-benzyne˙^+^ and ethynyl–methylene–cyclopropene˙^+^ have been performed at the B3LYP/6-311++G(2d,p) level of theory, whereas bicyclic *m*-benzyne˙^+^ has been calculated at the anharmonic B3LYP-GD3/N07D level of theory.

The experiments were performed with the FELion cryogenic ion trap apparatus^45^ stationed at the free-electron laser facility FELIX.^46^ Benzonitrile (99.9% for HPLC, Sigma-Aldrich) was evaporated at room temperature and the vapor was ionized by the impact of electrons. The formed HCN/HNC loss fragments with *m*/*z* 76 were used in this study as reactant species. Two different ion sources (temperature ∼300–400 K, pressures ∼10^−5^ mbar) were used: a direct electron impact (EI) ionization source and a Gerlich-type EI ion storage source^47^ yielding somewhat different isomeric ratios of the fragments. The species formed using the direct EI ion source have been previously spectroscopically characterized using infrared predissociation (IRPD) spectroscopy by Rap *et al.*^28^ The infrared spectrum from the direct EI ionization source was measured here at multiple EI energies of 16, 30 and 42 eV and averaged due to their similarity (Fig. 2a). The fragments formed upon impact of 17 eV electrons in the EI storage source were similarly identified in this study using IRPD (Fig. 2b).

For the kinetic measurements, *m*/*z* 76 fragment ions formed with EI energies of 17, 23 and 27 eV using both EI ionization sources, were mass selected by the first quadrupole mass spectrometer and guided into the 22-pole ion trap that was maintained at around 150 K. An intense (<15 ms long, number density of <10^15^ cm^−3^) helium pulse was admitted to the ion trap using a piezo valve to kinetically and internally cool down the ions. The trapped ions were exposed to a continuous flow of acetylene (Linde gas, 98%, with acetone solvent)-helium (various mixtures with typical ratios of 3 : 7, 3 : 17 and 3 : 18 of C_2_H_2_ : He) using a leakage valve to initiate and proceed the ion–molecule reaction for trapping, and thus reaction, times of 0–2600 ms. The formed products were detected by a second quadrupole mass spectrometer and a single ion detector (Daly detector). The acetylene number density was determined using the pressure reading of a hot-ionization gauge which was calibrated to a spinning rotor gauge directly connected to the ion trap. Two different ordinary differential equation (ODE) models were constructed to fit the experimental kinetic curves (see ESI† for details). A reduced ODE model that included one isomer and one reaction rate coefficient for the reaction of *m*/*z* 76 to *m*/*z* 102 was used in the low- and high-pressure regimes where the reaction is dominated by only one isomeric compound. A biexponential model was set up that included both a fast and slow reaction from *m*/*z* 76 to the intermediate species *m*/*z* 102. Pseudo first-order rates were determined by fitting the ODE model to the experimental kinetic curves as the acetylene number density is in significant excess compared to the number of ions. Second-order rate coefficients were obtained by a linear fit of the determined pseudo first-order rates at various acetylene number densities (Fig. S4, ESI†).

### Spectroscopic measurements of the reaction products

For the spectroscopic measurements, the *m*/*z* 76 reactants were formed in both the direct EI and storage EI ion source using an EI energy of 17 eV, where the isomeric ratios resemble the recorded infrared fingerprint spectra from IRPD measurements as shown in Fig. 2. To ensure the formation of the reaction products before the arrival of the first FEL pulse, the mass-selected *m*/*z* 76 species were reacted in the ion trap with a short (<15 ms, ∼10^−3^ mbar, ∼10^14^ cm^−3^) pulse of acetylene-helium (1 : 16.5 of C_2_H_2_ : He). The reaction intermediates and products were structurally characterized using infrared multiple photon dissociation (IRMPD) spectroscopy using the intense (up to 30 mJ) and tunable infrared radiation provided by the FEL-2 laser at the FELIX facility operating at 10 Hz in the 550–1700 cm^−1^ range with a FWHM of 0.5% of the center frequency. The FEL laser pulse structure typically consists of macro pulses of ∼10 μs filled with ∼3–6 ps long micro pulses each spaced by 1 ns. Resonant excitation of a vibrational mode led to fragmentation of the molecule and the amount of depletion of the respective target ion relative to the total number of ions without laser irradiation was measured as a function of wavelength to yield an infrared spectrum. As the ion cloud in the 22-pole ion trap is relatively large, generally up to 26 macro pulses were required to sample the trapped ions, and to observe a sufficient depletion signal. Calibration of the wavelength was performed using an infrared grating spectrum analyser and the signal was normalized to the laser pulse energy (*E*) and number of FELIX shots (*N*) to determine the relative cross section (*I*) according to:1
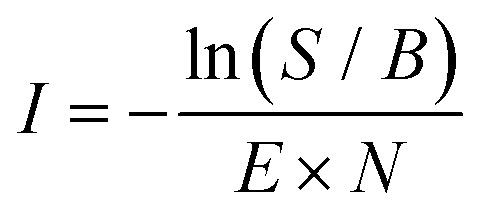
with *S* the observed ion counts and *B* the baseline ion count number.

### Quantum chemical calculations

The structures, electronic energies and the vibrational frequencies of the reactants, intermediates and product structures have been calculated with density functional theory (DFT) using Gaussian 16.^48^ The structures of the potential reaction products were optimized to their lowest energy structure using the B3LYP-GD3/N07D^49–51^ level of theory. Harmonic infrared frequencies of the structures were calculated at the B3LYP/6-311++G(2d,p) level of theory. For some structures, a better agreement with the experimental spectrum was obtained by including anharmonic contributions calculated using Second-order Vibrational Perturbation Theory (VPT2) at the B3LYP-GD3/N07D level of theory. Transition states were calculated at the B3LYP-GD3/N07D level of theory and verified using intrinsic reaction-coordinate (IRC) calculations. The electronic energies were corrected for the zero-point vibrational energy. The accuracy of the level of theory was established by comparison with transitions states calculated at the M06-2X/6-311++G(3df,2pd) level of theory which has been shown to predict accurate energies (Fig. S2, ESI†).^52–54^

The non-covalent interactions present for some of the reaction products have been calculated using NCI analysis^55^ with Multiwfn^56^ using the reduced electron density gradient:2
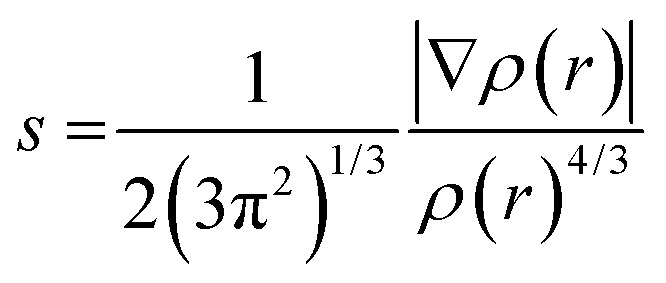
with *ρ* the electron density. A high-quality grid has been used for the calculation. An isosurface (0.6) of the reduced density gradient (*s*) was used and colored using the values of sign(*λ*_2_)*ρ*, with *λ*_2_ the second largest eigenvalue of the Hessian of the electron density, ranging from −0.03 to 0.02 (a.u.).

A value for the aromaticity was determined using the harmonic oscillator model of aromaticity (HOMA)^57^ implemented in Multiwfn^56^ and expressed as follows:3
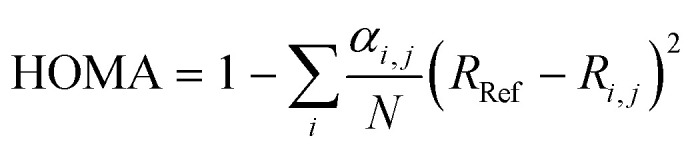
where *R*_*i*,*j*_ are the bond lengths of the molecule and *j* denotes the atom next to atom *i*, *N* is the number of atoms involved and *α*_*i*,*j*_ and *R*_Ref_ are constants from literature^57^ for each atom pair.

## Results

### Spectroscopic identification of the reactants

The structural characterization of the HCN/HNC loss fragments of benzonitrile is described in more detail by Rap *et al.* 2023,^28^ the experimental infrared spectra with the assigned structures are shown for completeness in Fig. 2a. Threshold photoionization and dissociation ionization of benzonitrile has been recently studied by Kamer *et al.* 2023^58^ and showed accurate dissociation barriers of the different fragmentation channels. Both *ortho*-benzyne˙^+^ and bicyclic *meta*-benzyne˙^+^ are identified as the fragments and are used here to perform controlled (mass-selected) reactions with acetylene molecules inside the 22-pole ion trap which was maintained at a temperature of around 150 K. No spectroscopic evidence of monocyclic *meta*-benzyne˙^+^ has been found and this structure is therefore not participating in the reaction with acetylene.

We have performed additional experiments where we have used an EI storage ion source which allows to collect and store the fragmentation products up to a few seconds, leading to better thermalization through collisional cooling and possibly chemical quenching due to secondary reactions. When measuring the experimental infrared spectrum of the fragment ions from this source (Fig. 2b), additional features are gaining intensity, indicating a third isomer with *m*/*z* 76. We can assign ethynyl–methylene–cyclopropene˙^+^ as the third isomer based on calculated vibrational bands that match the experimental bands (Fig. 2b and Fig. S1, ESI†). The features of this third isomer can also be identified in the direct source spectrum (Fig. 2a) but are relatively weak compared to the vibrational bands of the other isomers. A close inspection reveals a structure associated to ring-opening of (bicyclic) *m*-benzyne˙^+^. Likely, due to the longer timescale and/or the collisions that are characteristic of the ion storage source, some population of *m*-benzyne˙^+^ undergoes ring-opening to ethynyl–methylene–cyclopropene˙^+^, making its vibrational bands appear stronger in the recorded infrared spectrum.

### Kinetic measurements of the reactions

Having multiple isomers as reactants can complicate the experimentally observed chemistry. In order to disentangle the reactivity of the individual isomers, we use a combination of reaction kinetics, infrared action spectroscopy and quantum chemical calculations.

**Fig. 3:**
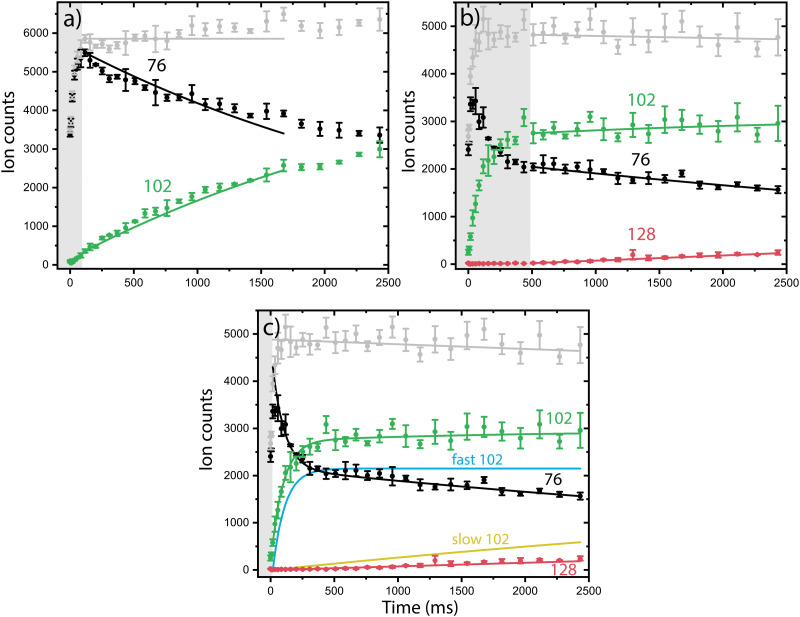
Representative kinetic plots of the *o*-benzyne˙^+^ (O)/*m*-benzyne˙^+^ (M)/ethynyl–methylene–cyclopropene˙^+^ (E) with acetylene ion–molecule reaction at 150 K for (a) low (1.36(±0.15) × 10^9^ cm^−3^) and (b) and (c) high (2.17(±0.23) × 10^10^ cm^−3^) acetylene number density. The experimental ion counts of the reactants (*m*/*z* 76, black), intermediate *m*/*z* 102 (green) and product structure *m*/*z* 128 (red) at different trapping times are plotted with dots and error bars. The data in panel a and b are fitted with the reduced ODE master equation whereas the data in panel c is fitted with the biexponential model (solid lines, explanation see text). The grey box indicates the data points that were not used in the fitting process.

Both isomeric mixtures as discussed in Fig. 2 have been used to initiate the ion–molecule reaction with acetylene, but we focus here first on the reactions of the mixture of *o*-benzyne˙^+^, bicyclic *m*-benzyne˙^+^ and ethynyl–methylene–cyclopropene˙^+^ as formed in the ion storage source. By varying the residence time of the mixture of ions with acetylene gas in the 22-pole ion trap, kinetic profiles can be obtained, as shown in Fig. 3. With a low acetylene number density (1.36(±0.15) × 10^9^ cm^−3^), the formation of an intermediate with *m*/*z* 102 is observed (Fig. 3a). When the acetylene number density is increased (2.17(±0.23) × 10^10^ cm^−3^), the formation of an additional secondary product with *m*/*z* 128 is observed (Fig. 3b). Moreover, one can clearly see a biexponential decay behavior of *m*/*z* 76, indicating the presence of at least two isomers reacting with significantly different reaction rate coefficients.

To establish the reaction rate coefficients, we have constructed a biexponential model using an ordinary differential equation (ODE) model that treats both a slow and fast reacting component of *m*/*z* 76, corresponding to two reacting (sets of) isomers (Fig. 3c, more detail in ESI†). An iterative approach was used varying the initial isomeric ratio by hand, and fitting the corresponding rate coefficients *k*_RA,fast_ and *k*_RA,slow_, for a set of acetylene number densities. The kinetic curves in both the lowest and highest acetylene number density regions can be assumed to be dominated by the reaction of only one isomeric compound and have additionally been simulated using a reduced ordinary differential equation (ODE) model including only one isomer and reaction rate coefficient (more details are given in ESI†).

The reaction rate coefficients of these low and high acetylene density measurements have been obtained from fitting the ODE model with the experimental data and are shown in Table S1 (ESI†). These values can be regarded as a lower limit for the fast reacting isomer (as the slower reacting isomer is contributing to some extend) and an upper limit for the slow reacting isomer (as some fast reacting isomer may still be present). Finally, we have combined both the single exponent fitted rate constants and the biexponentially fitted rate constants from both ion sources to yield accurate bimolecular reaction rate coefficients (using the known acetylene number densities) for the slow and fast reaction towards *m*/*z* 102. Additionally, the reaction to the product with *m*/*z* 128 from *m*/*z* 102 was included, assuming only one reacting isomer (Table 1) (Fig. S4, ESI†). No pressure dependence of the bimolecular reaction rate coefficient has been observed, therefore we can conclude that the determined rate coefficients for the reactions to *m*/*z* 102 and *m*/*z* 128 are dominated by radiative association where the excess energy of the reaction is removed upon the release of photons, *i.e.*, we are not operating in a pressure regime where termolecular processes are important (Fig. S5, ESI†).

**Table 1:** Experimentally determined bimolecular reaction rate coefficients of the ion–molecule reaction of *o*-benzyne˙^+^ (O)/*m*-benzyne˙^+^ (M)/ethynyl–methylene–cyclopropene˙^+^ (E) with acetylene

Reaction	Rate coefficient (cm^3^ s^−1^)	Type	Label	Number density range C_2_H_2_ (cm^−3^)
76˙^+^ → 102˙^+^ (fast)	3.9(±0.2) × 10^−10^	Radiative association	*k*_RA,fast_	1.0 × 10^9^–8.2 × 10^10^
76˙^+^ → 102˙^+^ (slow)	2.1(±0.3) × 10^−12^	Radiative association	*k*_RA,slow_	3.4 × 10^9^–2.9 × 10^11^
102˙^+^ → 128˙^+^	1.5(±0.2) × 10^−12^	Radiative association	*k*_RA,C10_	3.4 × 10^9^–2.9 × 10^11^

The fast reacting isomer with *m*/*z* 102 is determined to have a bimolecular rate coefficient of 3.9(±0.2) × 10^−10^ cm^3^ s^−1^, whereas the other isomer reacts more than two orders of magnitude (∼186 ± 28) slower with a determined bimolecular reaction rate coefficient of 2.1(±0.3) × 10^−12^ cm^3^ s^−1^. The fast reaction to *m*/*z* 102 is slightly slower than an analogues ion–molecule reaction of phenyl^+^ (C_6_H_5_^+^) and acetylene to *m*/*z* 103 at a bimolecular rate coefficient of 7.1(±1.4) × 10^−10^ cm^3^ s^−1^ (at 305 K).^14^ Also, the reaction towards *m*/*z* 128 is determined to be of the same order of magnitude as the slow reaction towards *m*/*z* 102 with a bimolecular rate coefficient of 1.5(±0.2) × 10^−12^ cm^3^ s^−1^. This value must be regarded as a lower limit since only one isomer with *m*/*z* 102 is likely to react to *m*/*z* 128, which is not explicitly treated in the ODE model.

Using the isomers formed in the direct ion source, which are predominantly *o*-benzyne˙^+^ (O) and *m*-benzyne˙^+^ (M), we have tried to get more information on which isomers are responsible for the slow and fast reaction rate coefficients as determined. Representative kinetic plots measured at high acetylene density using either the direct ion source or ion storage source show crucial differences (Fig. S3, ESI†). In both panels of Fig. S3 (ESI†), the *m*/*z* 76 curve shows a flattening indicating that the faster isomer has almost completely reacted away, but, in each plot, the plateau is positioned differently relative to the total ion count. This indicates that more of a slower reacting *m*/*z* 76 isomer is present for the ion storage source reactants and hints to ethynyl–methylene–cyclopropene˙^+^ being this slower component as it is more pronounced in the infrared spectrum of the ions generated in the ion storage source (Fig. 2).

### Spectroscopic probing of the reaction products

**Fig. 4:**
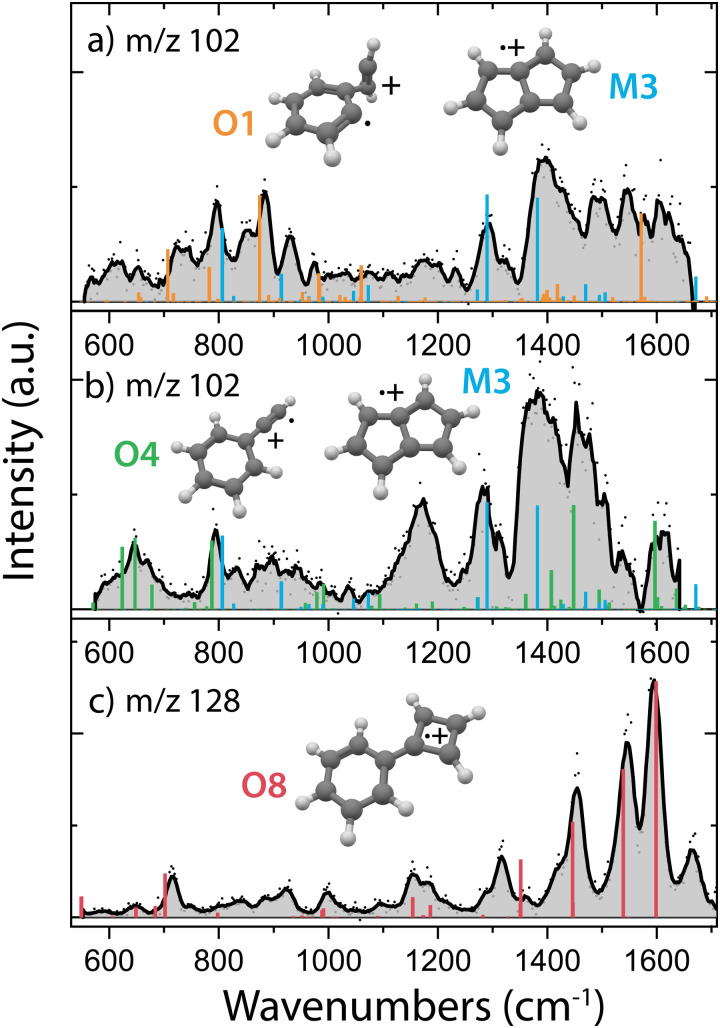
Experimental infrared fingerprint spectra (grey) of the (a) and (b) *m*/*z* 102 and (c) *m*/*z* 128 reaction intermediates and products from the ion–molecule reaction of *o*-benzyne˙^+^ (O)/*m*-benzyne˙^+^ (M)/ethynyl–methylene–cyclopropene˙^+^ (E) with acetylene. Two different spectra are shown for *m*/*z* 102 corresponding to experimental conditions where (a) no *m*/*z* 128 is formed and (b) the product with *m*/*z* 128 is formed. Calculated anharmonic infrared frequencies at the B3LYP-GD3/N07D level of theory of the assigned dominant structures are shown as sticks for (a) covalently bound acetylene *ortho*-benzyne˙^+^ complex (O1, orange) and pentalene˙^+^ (M3, blue) and (b) phenylacetylene˙^+^ (O4, green) and pentalene˙^+^ (M3, blue). Calculated harmonic vibrational modes at the B3LYP/6-311++G(2d,p) level of theory for phenylcyclobutadiene˙^+^ (O8, red) are shown in panel (c).

In order to obtain the structural information of the formed *m*/*z* 102 and *m*/*z* 128 species, we have recorded their infrared fingerprint spectra using Infrared Multiple Photon Dissociation (IRMPD) spectroscopy using the free electron laser FELIX^46^ (Fig. 4), as described in more detail in the Methods. We have used the isomeric composition from the ion storage source for the *m*/*z* 76 reactants (Fig. 2b) and added acetylene in a diluted high-pressure pulse at the beginning of the trapping cycle, so that the reaction is terminated before the spectroscopic probing.

To obtain more information on the important reaction intermediate (*m*/*z* 102) for the reaction to *m*/*z* 128, we have measured the infrared fingerprint spectrum of the intermediate *m*/*z* 102 for two different experimental conditions: one where the reaction has been tuned to only observe the intermediate *m*/*z* 102 (Fig. 4a) and the other where also the product *m*/*z* 128 is formed (Fig. 4b). This has been achieved by using a three times lower acetylene number density for the measurements of the here so-called interrupted reaction (Fig. 4a), therefore we are predominantly observing the fast reaction to *m*/*z* 102 compared to the here so-called proceeded reaction (Fig. 4b) with a higher acetylene number density where the product with *m*/*z* 128 is formed.

Specific molecular structures were assigned to the experimental bands by comparison with calculated (an)harmonic infrared frequencies. We clearly identify features characteristic of pentalene˙^+^ (M3) around 1295 cm^−1^ and 1395 cm^−1^ (C–H in plane bending) in both the spectra of *m*/*z* 102 taken with (Fig. 4b) and without (Fig. 4a) observing the end product with *m*/*z* 128. This means that the reaction towards pentalene˙^+^ is fast and contributes to the bimolecular rate constant that we have determined to be 3.9(±0.2) × 10^−10^ cm^3^ s^−1^. Moreover, some vibrational features disappear and others appear upon the proceeded reaction. Characteristic signatures of a covalently bound acetylene *ortho*-benzyne˙^+^ complex (O1) are visible around 885 cm^−1^ (C–H bending of acetylene group) in the spectrum of the interrupted reaction (Fig. 4a) whereas they are absent in the spectrum of the proceeded reaction (Fig. 4b). Instead, vibrational modes belonging to phenylacetylene˙^+^ (O4) are present in the latter. Two weak fundamental C–H in plane bending modes of phenylacetylene˙^+^ can explain the experimental feature around 1170 cm^−1^, although their intensity is theoretically underpredicted.

The structure of *m*/*z* 128 can be assigned to phenylcyclobutadiene˙^+^ (O8), a somewhat surprising structure consisting of a four and a six membered ring linked with a covalent bond, based on a good agreement between the experimental and theoretical spectrum. The three strongest experimental features at 1455, 1544 and 1595 cm^−1^ are well explained by calculated vibrational modes of phenylcyclobutadiene˙^+^. The experimental band at 1316 cm^−1^ can be explained by CC stretching vibrations of the four-membered ring, however the predicted band position is significantly shifted to 1360 cm^−1^. A lower abundance limit of 80% can be obtained from the relative depletion value of the most intense band of *m*/*z* 128 shown in Fig. S9 (ESI†) establishing phenylcyclobutadiene˙^+^ as the major product.

As the second reaction to *m*/*z* 128 is experimentally determined to be slow (1.5(±0.2) × 10^−12^ cm^3^ s^−1^, Table 1), and since we observe *m*/*z* 128 under the same experimental conditions as used for probing the proceeded spectrum of *m*/*z* 102 (Fig. 4b), we can assume that this intermediate is still likely present in the spectrum, in particular since the first reaction step from *m*/*z* 76 to *m*/*z* 102 is determined to be much faster (3.9(±0.2) × 10^−10^ cm^3^ s^−1^, Table 1). Both pentalene˙^+^ and phenylacetylene˙^+^ are detected and may therefore play a role as reaction intermediate.

Surprisingly, the formation of the better known and more stable naphthalene˙^+^ (NI3) is not spectroscopically observed here, whereas it has been previously proposed as the product upon the ion–molecule reaction of phenylacetylene˙^+^ and acetylene.^59^ For both the direct and storage ion source, we have measured a similar infrared spectrum of the product with *m*/*z* 128 (Fig. S10, ESI†), which indicates that ethynyl–methylene–cyclopropene˙^+^ (E), which is practically absent amongst the direct ion source reactants (Fig. 2a), is not the important reactant for this product.

### Potential energy surfaces of the reaction to *m*/*z* 102

**Fig. 5:**
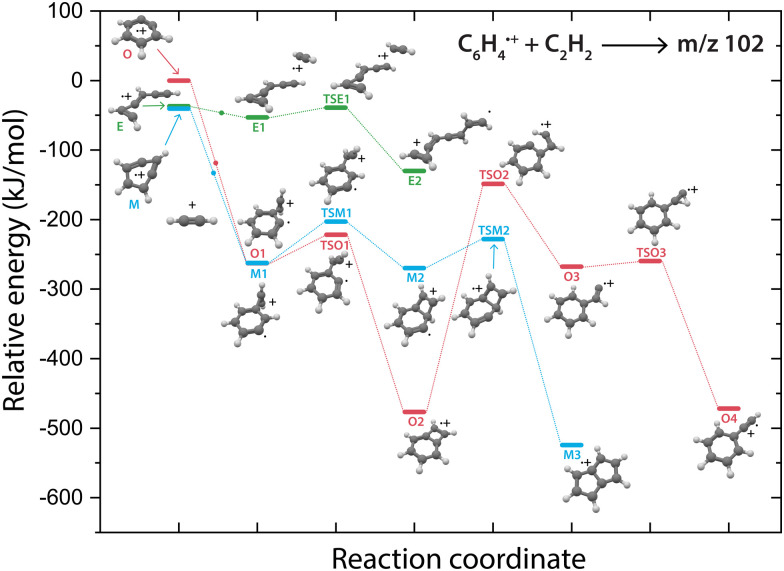
Potential energy surface of the acetylene addition reaction of *o*-benzyne˙^+^ (O, red pathway), *m*-benzyne˙^+^ (M, blue pathway) and ethynyl–methylene–cyclopropene˙^+^ (E, green pathway). The small dots along the pathway display the addition of an acetylene molecule. The energies are calculated at the B3LYP-GD3/N07D level of theory and are corrected for the zero-point vibrational energy.

Overall, there are three isomeric species with *m*/*z* 76 that may react and we observe both a fast and slow reaction towards the multiple structures detected in the *m*/*z* 102 channel. To get more insight into which isomer is responsible for which intermediate, we have performed potential energy surface (PES) calculations of the acetylene addition to all three *m*/*z* 76 isomers (Fig. 5).

The addition of acetylene to *o*-benzyne˙^+^ and bicyclic *m*-benzyne˙^+^ forms a covalently bound complex with a triangular arrangement of the acetylene group, existing at 263 kJ mol^−1^ (O1) and 222 kJ mol^−1^ (M1) lower in energy than their corresponding reactants, respectively. Contrarily, ethynyl–methylene–cyclopropene˙^+^ forms a non-covalently bound complex with acetylene which only exists 16 kJ mol^−1^ (E1) lower in energy and is not spectroscopically observed. This may give an indication that the latter reaction proceeds with slower reaction rate coefficient. Also, the formation of a covalent bond *via*TSE1, existing at −2 kJ mol^−1^, is energetically unfavorable.

We may not be able to disentangle the two reactions of *o*-benzyne˙^+^ and bicyclic *m*-benzyne˙^+^ with acetylene which are both expected to proceed fast based on the calculated energetics. Interestingly, from the covalently bound acetylene-*m*-benzyne˙^+^ complex (M1), the bicyclic pentalene˙^+^ structure (M3), identified in the infrared spectrum of the *m*/*z* 102 intermediate, can be readily formed *via* ring-closure (TSM1) and skeletal rearrangement (TSM2). Similarly, ring-closure (TSO1) from the analogues covalently bound acetylene-*o*-benzyne˙^+^ complex (O1) forms the energetically favorable benzocyclobutadiene˙^+^ (O2) structure. Surprisingly, we cannot assign all the vibrational modes of benzocyclobutadiene˙^+^ to experimentally observed infrared features (Fig. S6 and S7, ESI†), meaning that this isomer is not formed (at high abundance). A higher lying transition state, however, involving ring-opening (TSO2) and subsequent hydrogen migration is calculated and leads to the spectroscopically observed phenylacetylene˙^+^ (O4).

The covalently bound acetylene *ortho*-benzyne˙^+^ complex (O1) is experimentally observed in the interrupted spectrum of *m*/*z* 102 (Fig. 4a), whereas phenylacetylene˙^+^ (O4) is observed in the proceeded spectrum of *m*/*z* 102 (Fig. 4b). It is likely that O1 isomerizes rapidly into O4 without sampling O2. This may also occur *via* a direct pathway from O1 to O4 upon a hydrogen migration process from the acetylene group to the ring, however, we were not able to locate the corresponding transition state on the PES. A competing process to isomerization is the stabilization and trapping of O1 by collisions with He in the initial high-density pulse. This process seems to be enhanced during the spectroscopic probing of the interrupted spectrum (Fig. 4a), which had a higher He partition, and shows a higher abundance of O1. A similar effect has been observed previously, where the intermediate complex of a bimolecular ion–molecule reaction was trapped and investigated.^60^

### Potential energy surface of the reaction to *m*/*z* 128

**Fig. 6:**
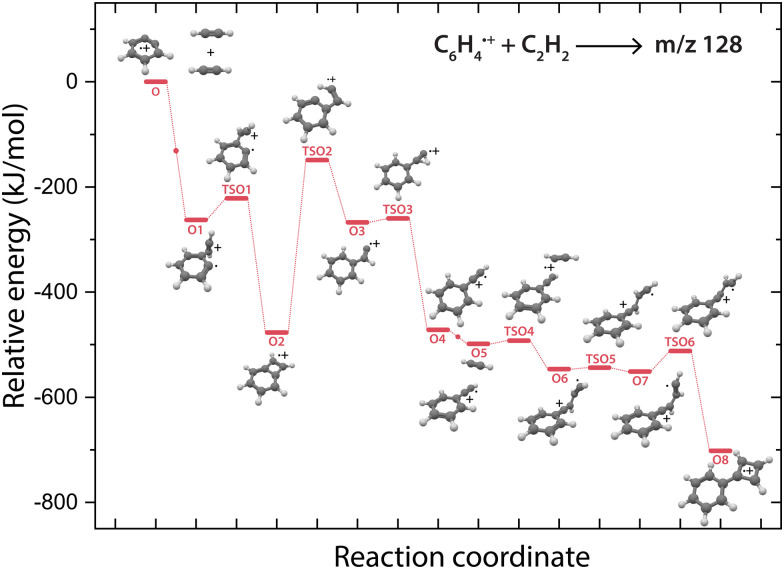
Potential energy surface of the reaction of *o*-benzyne˙^+^ (O) with acetylene towards the *m*/*z* 128 product phenylcyclobutadiene˙^+^ (O8, red pathway). The small dots along the pathway display the addition of a new acetylene molecule. The energies are calculated at the B3LYP-GD3/N07D level of theory and are corrected for the zero-point vibrational energy.

The structure of the assigned final product phenylcyclobutadiene˙^+^ (O8) and the detection of phenylacetylene˙^+^ (O4) hints towards the latter as the involved reaction intermediate for the secondary reaction. To investigate this, a PES towards phenylcyclobutadiene˙^+^*via* phenylacetylene˙^+^ has been calculated (Fig. 6).

The addition of a second acetylene molecule proceeds *via* the formation of a non-covalently bound complex (O5). The reaction continuous *via* the addition at the end of phenylacetylene˙^+^ to form a C_4_H_3_ chain (O7) that can close to form a four-membered ring *via*TSO6. The latter part of this pathway occurs similar as observed for the ion–molecule reaction of benzonitrile˙^+^ and acetylene,^61^ also displaying the formation of a non-covalently bound complex upon the addition of the second acetylene molecule and the formation of a C4 carbon chain that can form an additional covalently linked ring.

### Non-covalent interactions of the reaction intermediates to *m*/*z* 128

The question arises why the formation of naphthalene˙^+^ is not observed experimentally. Both the comparison with calculated vibrational modes of naphthalene˙^+^ (Fig. S8, ESI†) and with an experimental reference spectrum (Fig. S9, ESI†) provide no evidence of its formation. Interestingly, earlier kinetic studies of the ion–molecule reaction of phenylacetylene˙^+^ and acetylene also showed the formation of an ion with *m*/*z* 128 at a similar rate coefficient of 1.5(±1.1) × 10^−12^ cm^3^ s^−1^ (at 302 K),^59^ and the authors suggested that this product is naphthalene˙^+^ based on its low energy and its stability observed in the experiment. The spectroscopic identification of phenylcyclobutadiene˙^+^ as performed here adds crucial information and strengthens the idea that phenylacetylene˙^+^ is the important intermediate in the reaction towards phenylcyclobutadiene˙^+^.

**Fig. 7:**
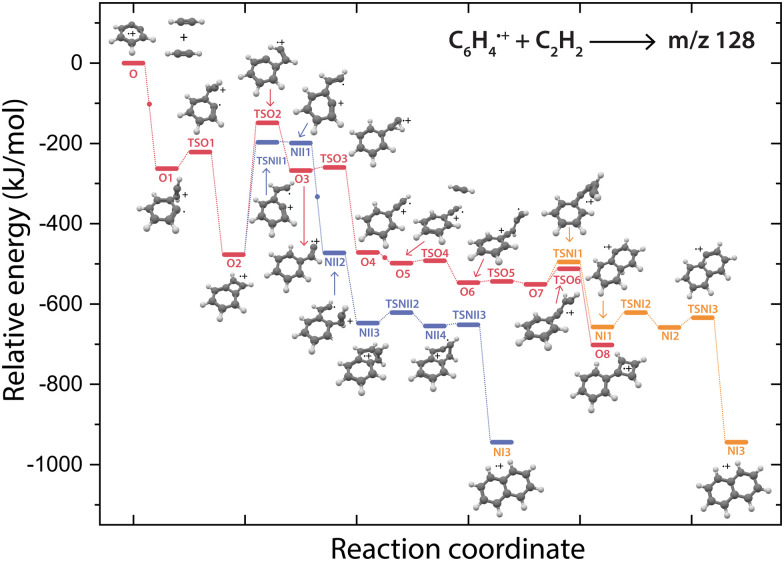
Potential energy surfaces of the reaction of *o*-benzyne˙^+^ (O) with acetylene towards the product with *m*/*z* 128 phenylcyclobutadiene˙^+^ (O8, red pathway) and naphthalene˙^+^ (NI3, purple and orange pathways). The small dots along the pathway display the addition of a new acetylene molecule. The energies are calculated at the B3LYP-GD3/N07D level of theory and are corrected for the zero-point vibrational energy.

**Fig. 8:**
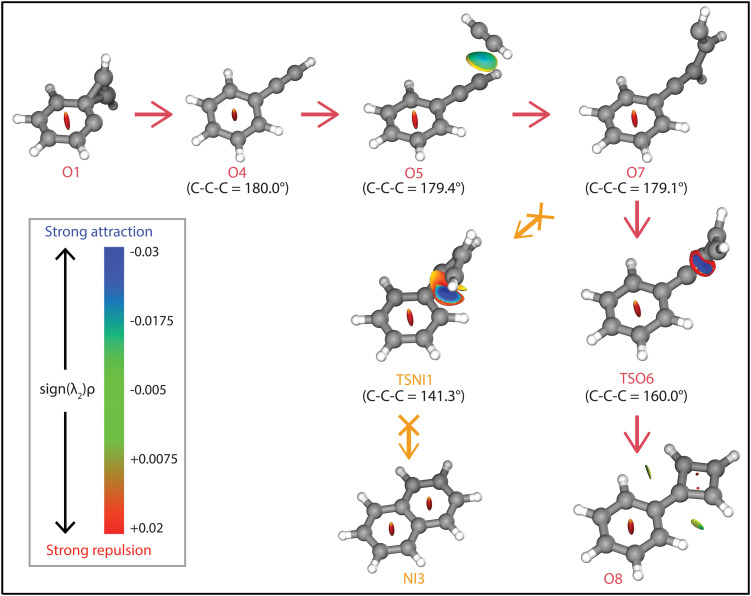
NCI plots of intermediates and transition states of the reaction pathways towards phenylcyclobutadiene˙^+^ (O8, red arrows), and naphthalene˙^+^ (NI3, orange arrows). The strengths of the NCIs are shown by the color spectrum ranging from red (strong repulsion), green (weak attraction) and blue (strong attraction). The calculated angle of the C–C–C group is shown next to the structures.

To investigate this surprising finding, two potential pathways from *o*-benzyne˙^+^ towards naphthalene˙^+^ have been calculated and are compared to the formation of phenylcyclobutadiene˙^+^ in Fig. 7. To aid the comparison, the non-covalent interactions (NCIs) of important reaction intermediates have been calculated using Multiwfn^56^ (Fig. 8, Fig. S11, ESI†).

The addition of acetylene and ring-closure (TSO1) to benzocyclobutadiene˙^+^ and subsequent ring-opening (TSO2) reactions to phenylacetylene˙^+^ are likely fast and proceed prior to the arrival of a second acetylene molecule. Therefore, a pathway where phenylacetylene˙^+^ reacts with another acetylene is more probable. The reaction proceeds with a second acetylene addition and finally ring-closure to form phenylcyclobutadiene˙^+^ (O8). The addition of acetylene on the ethynyl chain of phenylacetylene˙^+^ (O4) is favored by a weak attractive non-covalent interaction as shown as green/blue disks in Fig. 8.

Two diverging pathways to naphthalene˙^+^ have been located on the PES towards phenylcyclobutadiene˙^+^. A ring-opening transition state (TSNII1), analogues to TSO2, from benzocyclobutadiene˙^+^ exists and forms an intermediate structure (NII1) that is susceptible for a second acetylene to attach. Further steps to naphthalene˙^+^ include the formation of a five-membered and three-membered ring moiety (NII4) that can expand to the six-membered ring naphthalene˙^+^ structure. The acetylene-*o*-benzyne˙^+^ intermediate (NII1), however, has a comparable energy as the accompanying transition state (TSNII1) which likely enables recrossing to the energetically more favorable structure benzocyclobutadiene˙^+^ (O2) prior to the addition of a second acetylene molecule. This is also supported by the visualized non-covalent interactions of the transition state (TSNII1) (Fig. S11, ESI†), where a weak attractive (vdW) interaction is present between the acetylene group and the carbon atom on the ring. This favors the backwards reaction by steering a correct geometry for ring-closure instead of rotation of the acetylene group.

At the end of the pathway to phenylcylcobutadiene˙^+^, we can again diverge *via* a different transition state (TSNI1) towards naphthalene˙^+^. An analogues transition state where the C_4_H_3_ group undergoes ring closure, now at the carbon atoms of the ring, forms a bicyclic six-membered ring structure (NI1) that only requires hydrogen migration steps to form naphthalene˙^+^. The NCIs of both ring-closing transition states TSNI1 and TSO6 are visualized in Fig. 8 and exhibit a blue/red disk that indicates a strong attractive interaction in the middle and a repulsive character at the edges of the disk. Similar non-covalent interactions have been observed in NCI calculations of other transitions states.^62,63^ The NCIs show that both transition states are accessible in a confined space. Moreover, in case of the transition state to naphthalene˙^+^, the sp hybridized C–C–C bond is heavily distorted measuring an angle of 141.3° compared to 160.0° in the analogues transition state to phenylcyclobutadiene˙^+^. This can be a measure of the flexibility that is required to proceed *via* these transition states, making the formation of naphthalene˙^+^ less probable than phenylcyclobutadiene˙^+^.

## Astrophysical implications and conclusions

By separating the fragmentation and recombination processes, the complex chemistry of reactive ionic fragments of benzonitrile with acetylene has been revealed. Laboratory studies using EI dissociative ionization showed the formation of *o*-benzyne˙^+^ and bicyclic *m*-benzyne˙^+^ as the HCN/HNC loss product.^28^ The combination of kinetics, infrared spectroscopy and quantum chemical calculations used here has revealed the reactivity of both these fragments, *o*-benzyne˙^+^ and bicyclic *m*-benzyne˙^+^, with acetylene and showed the formation of potentially astronomically relevant species such as pentalene˙^+^, phenylacetylene˙^+^ and phenylcyclobutadiene˙^+^. Surprisingly, we observe only association and no bimolecular reactions which is likely due to the relatively hydrogen-poor reactant molecules. The formation of phenylcyclobutadiene˙^+^ as the product of two acetylene additions to *o*-benzyne˙^+^, instead of the lower energy and more commonly discussed naphthalene, shows that structural characterization of the reaction products using spectroscopic methods is extremely important in order to assign the correct product. The spectroscopic proof of the bicyclic covalently linked product emphasizes that this class of molecules can be important for interstellar chemistry compared to the often-studied fused PAHs such as naphthalene.^61^

The studied system is astrochemically relevant as both neutral benzonitrile^4^ and *o*-benzyne^64^ have been detected in the cold molecular cloud TMC-1. Upon UV photo-ionization or cosmic ray ionization the major HCN/HNC loss channel of benzonitrile, which has appearance energies reported in a range between 13.1 eV (ionization energy to cationic ground state + rate-determining HCN-loss barrier)^58^ and 13.38 eV,^65^ is accessible and reactive species can be formed. The chemistry of neutral *o*-benzyne with the small hydrocarbon allyl radical has previously been studied using double imaging photoelectron photoion coincidence spectroscopy (i^2^PEPICO) and showed the formation of the bicyclic molecule indene.^11^ Also, the reaction of *o*-benzyne with methyl radical showed multiple five-membered ring species that have been recently detected in TMC-1.^66^ We have demonstrated that also the cationic *o*-benzyne˙^+^ reacts efficiently with the small hydrocarbon acetylene *via* radiative association (3.9(±0.2) × 10^−10^ cm^3^ s^−1^). Moreover, an efficient pathway from *m*-benzyne˙^+^ to the spectroscopically detected pentalene˙^+^ structure is calculated. The pentagonal motif is also observed in fragmentation products of (N)PAHs^23,25,67,68^ and multiple five-membered ring structures have been detected in TMC-1.^69–72^

Neutral pentalene, however, exhibits an antiaromatic 8π electron system. We can obtain an estimate on the (anti)aromaticity of the products using a simple model called the harmonic oscillator model of aromaticity (HOMA)^57^ that treats the so-called geometrical indices of aromaticity, which are the bond-lengths compared to the bond-lengths of a system with a full π-electron delocalization (Table S2, ESI†). Whereas the neutral pentalene structure exhibits a strong antiaromatic character, the radical cationic structure has an overall aromatic character. Similarly, the cyclobutadiene moiety of neutral phenylcyclobutadiene is strongly antiaromatic. However, cationic phenylcyclobutadiene˙^+^ shows a non-aromatic cyclobutadiene moiety and an aromatic phenyl group. More detail on the aromaticity should be obtained by investigating other criteria of aromaticity such as magnetic properties, reactivity and especially the effect of the conjugated rings in the case of phenylcyclobutadiene˙^+^.^73^ Altogether, the radical cationic forms of pentalene and phenylcyclobutadiene have increased aromaticity upon the removal of an electron from their neutral antiaromatic 8π and 10π electron systems, respectively,^74^ and thus a higher stability and likelihood to survive in the ISM.

Finally, the destruction and recombination processes may yield increasingly stable species that can survive longer in parts of the ISM where energetic processing takes place, *e.g.*, PDRs, and are less susceptible to further chemical reactions. This will influence the observed astronomical abundances and needs to be correctly implemented into the astrochemical modeling. By separating both fragmentation and formation reactions in both laboratory experiments and theoretical approaches, as performed in this study, required parameters for such a model, *e.g.*, rate coefficients and structural information can be obtained. Furthermore, the obtained IR spectra of the product ions can be used as fingerprints to interpret astronomical infrared spectra from JWST.^75^ The small ion CH_3_^+^ has recently been detected in a protoplanetary disk^76^ and larger ionic molecules could be detected by comparison with laboratory data and theoretical models.^77^

## Author contributions

D. B. R.: conception and design of work, data collection, data analysis and interpretation, theoretical calculations, visualization, drafting and editing of article. J. G. M. S.: data collection, data analysis and interpretation, editing of article. B. R.: funding acquisition, project administration, supervision, data interpretation, editing of article. S. B.: conception and design of work, data collection, data interpretation, methodology, project administration, funding acquisition, supervision, drafting and editing of article. All authors discussed the manuscript.

## Conflicts of interest

The authors declare no competing interests.

## Supplementary Material

CP-026-D3CP05574D-s001
