# Supplementary material for: Ionic fragmentation products of benzonitrile as important intermediates in the growth of polycyclic aromatic hydrocarbons
Source: Phys Chem Chem Phys. 2024 Feb 6;26(9):7296–307. doi: 10.1039/d3cp05574d (PMC10900304; doi:10.1039/d3cp05574d)
Supplement: CP-026-D3CP05574D-s001 [file CP-026-D3CP05574D-s001.pdf]

Electronic Supplementary Material (ESI) for Physical Chemistry Chemical Physics.
This journal is © the Owner Societies 2024

Supplementary Information:

Ionic Fragmentation Products of Benzonitrile as Important Intermediates in the Growth of Polycyclic Aromatic Hydrocarbons

Daniël B. Rap, Johanna G.M. Schrauwen, Britta Redlich and Sandra Brünken*

Radboud University, FELIX Laboratory, Institute for Molecules and Materials, Toernooiveld 7, 6525 ED Nijmegen, The Netherlands, *E-mail: sandra.bruenken@ru.nl

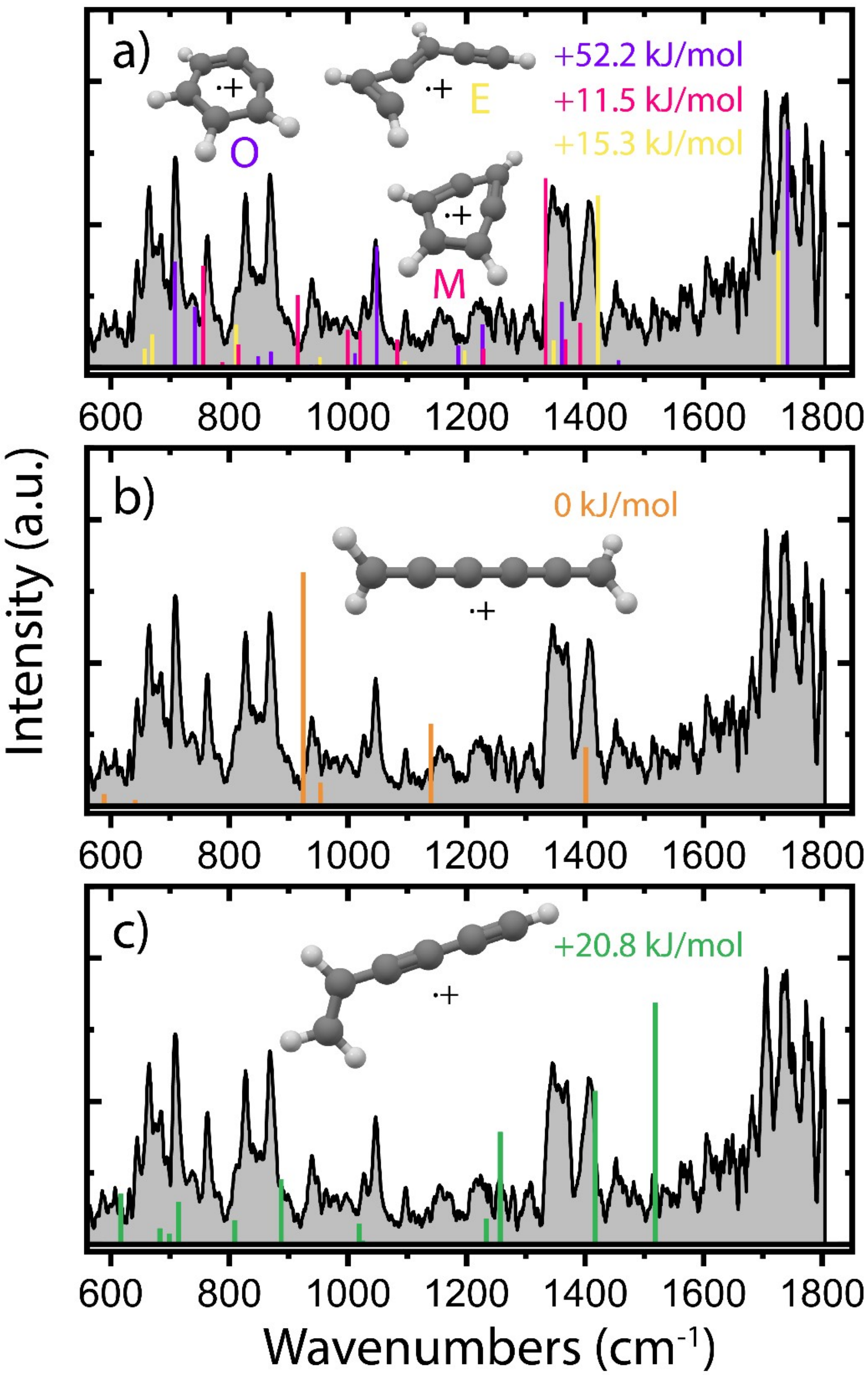

Supplementary Fig. 1: Experimental infrared fingerprint spectra of the reactants with m/z 76 formed in the ion storage source (grey). Calculated vibrational modes are shown for (a) the assigned o-benzyne$^{\bullet+}$ (**O**, purple), bicyclic m-benzyne$^{\bullet+}$ (**M**, pink) and ethynyl-methylene-cyclopropene$^{\bullet+}$ (**E,** yellow), (b) linear $H_2C_6H_2^{\bullet+}$ (orange) and (c) vinyl-diacetylene$^{\bullet+}$ (green). The calculations have been performed at the harmonic B3LYP-GD3/N07D level of theory. The zero-point vibrational energy corrected electronic energies of the molecules are shown relative to the energy of the lowest energy structure.

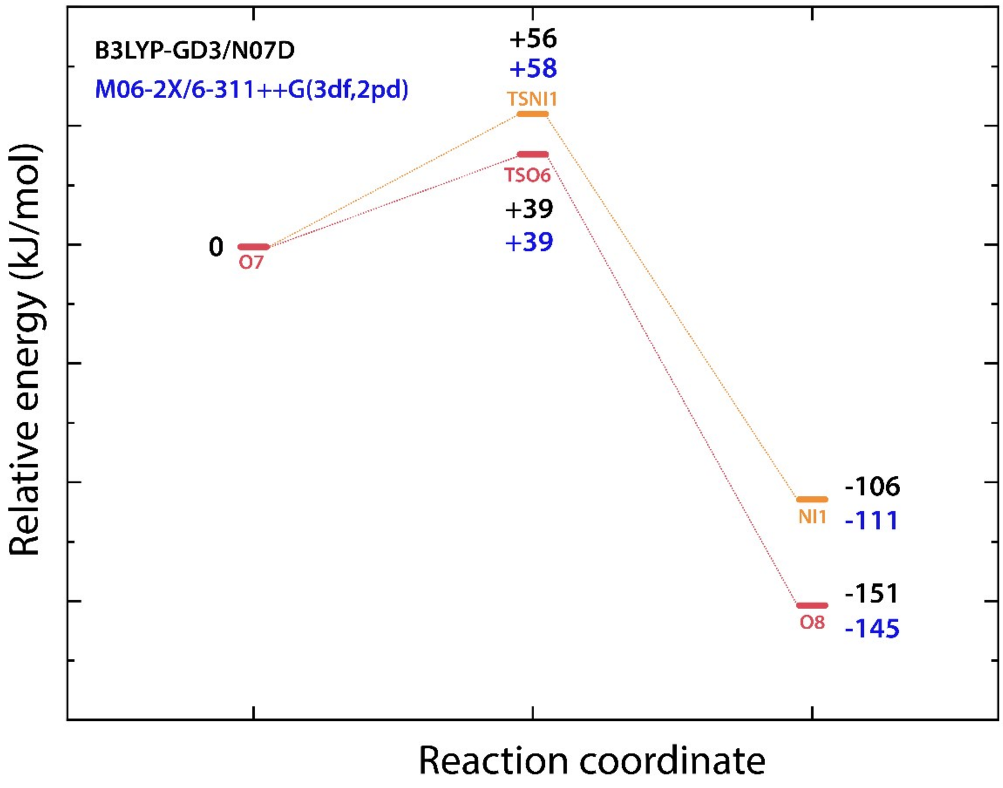

Supplementary Fig. 2: Relative energies of the transition states TSNI1 and TSO6 calculated at the B3LYP-GD3/N07D (black) and M06-2X/6-311++G(3df,2pd) (blue) level of theory. The energies are corrected for the zero-point vibrational energy.

Fitting the kinetic curves with an ODE model

Two ODE models have been constructed that describe the observed reactions and are shown below. In the reduced model, only one reaction towards m/z 102 is assumed. For the biexponential model, the decay of m/z 76 was simulated using a fast and slow component. A starting ratio between the fast and slow isomer for each ion source was estimated based on the kinetic curves (**Supplementary Fig. 2**). Starting ratio values of 2.4 (~70 % fast isomer) and 1 (50 % fast isomer) were used for the direct ion source and storage ion source, respectively. The ODE models are solved for a pseudo-first-order approximation and the solutions are fitted to the measured data to yield the first-order rate coefficients (s^{-1}). The second-order rate coefficients are determined by plotting the first-order rate against the acetylene number density as shown in **Supplementary Fig. 3**. A linear fit of the data yields the second-order rate coefficient of the reaction. The resulting reaction rate coefficients obtained using the individual models and the fitted results of both models combined are shown in **Supplementary Tab. 1.**

Reduced ODE model:

$$\frac{d[C_6H_4{}^{\bullet+}]}{dt} = -\, k_{RA} * [C_6H_4{}^{\bullet+}]_{total} * [C_2H_2]$$

$$\frac{d[C_8H_6{}^{\bullet+}]}{dt} = +\, k_{RA} * [C_6H_4{}^{\bullet+}]_{total} * [C_2H_2]$$

$$-\, k_{RA,C10} * [C_8H_6{}^{\bullet+}] * [C_2H_2]$$

$$\frac{d[C_{10}H_8{}^{\bullet+}]}{dt} = +\, k_{RA,C10} * [C_8H_6{}^{\bullet+}] * [C_2H_2]$$

Biexponential ODE model:

$$\frac{d[C_6H_4{}^{\bullet+}]}{dt} = -\, k_{RA,fast} * [C_6H_4{}^{\bullet+}]_{total} * \frac{ratio}{1+ratio} * [C_2H_2]$$

$$-\, k_{RA,slow} * [C_6H_4{}^{\bullet+}]_{total} * \frac{1}{1+ratio} * [C_2H_2]$$

$$\frac{d[C_8H_6{}^{\bullet+}]}{dt} = +\, k_{RA,fast} * [C_6H_4{}^{\bullet+}]_{total} * \frac{ratio}{1+ratio} * [C_2H_2]$$

$$+\, k_{RA,slow} * [C_6H_4{}^{\bullet+}]_{total} * \frac{1}{1\,+\,ratio} * [C_2H_2]$$

$$-\, k_{RA,C10} * [C_8H_6{}^{\bullet+}] * [C_2H_2]$$

$$\frac{d[C_{10}H_8{}^{\bullet+}]}{dt} = +\, k_{RA,C10} * [C_8H_6{}^{\bullet+}] * [C_2H_2]$$

$$\frac{d\,ratio}{dt} = -\left(k_{RA,fast} - k_{RA,slow}\right) * ratio$$

With:

$$[C_6H_4{}^{\bullet +}]_{total} = [C_6H_4{}^{\bullet +}]_{fast} + [C_6H_4{}^{\bullet +}]_{slow} \quad \text{and} \quad ratio = \frac{[C_6H_4{}^{\bullet +}]_{fast}}{[C_6H_4{}^{\bullet +}]_{slow}}$$

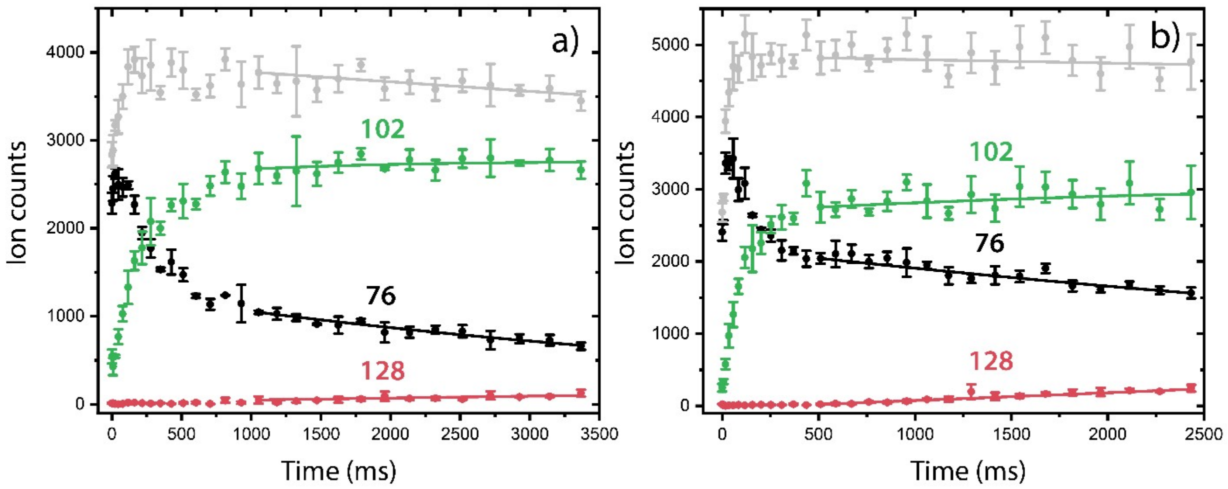

Supplementary Fig. 3: Representative kinetic plots of the o-benzyne•+ (**O**)/m-benzyne•+ (**M**)/ethynyl-methylene-cyclopropene•+ (**E**) with acetylene ion-molecule reaction using (a) the direct ion source and (b) the ion storage source at acetylene number densities of 3.4(±0.4) · 10^9 cm^{-3} and 2.17(±0.23) · 10^{10} cm^{-3}, respectively. The experimental ion counts of the reactants (m/z 76, black), intermediate m/z 102 (green), product structure m/z 128 (red) and total ion count (grey) at different trapping times are plotted with dots and error bars. The kinetic plots in panel a and b have been fitted in a regime where we assume that the fast isomer has almost completely reacted away using the reduced ODE model that threats the reaction from m/z 76 to m/z 102 with one rate coefficient. The results of the fit are plotted with a solid line.

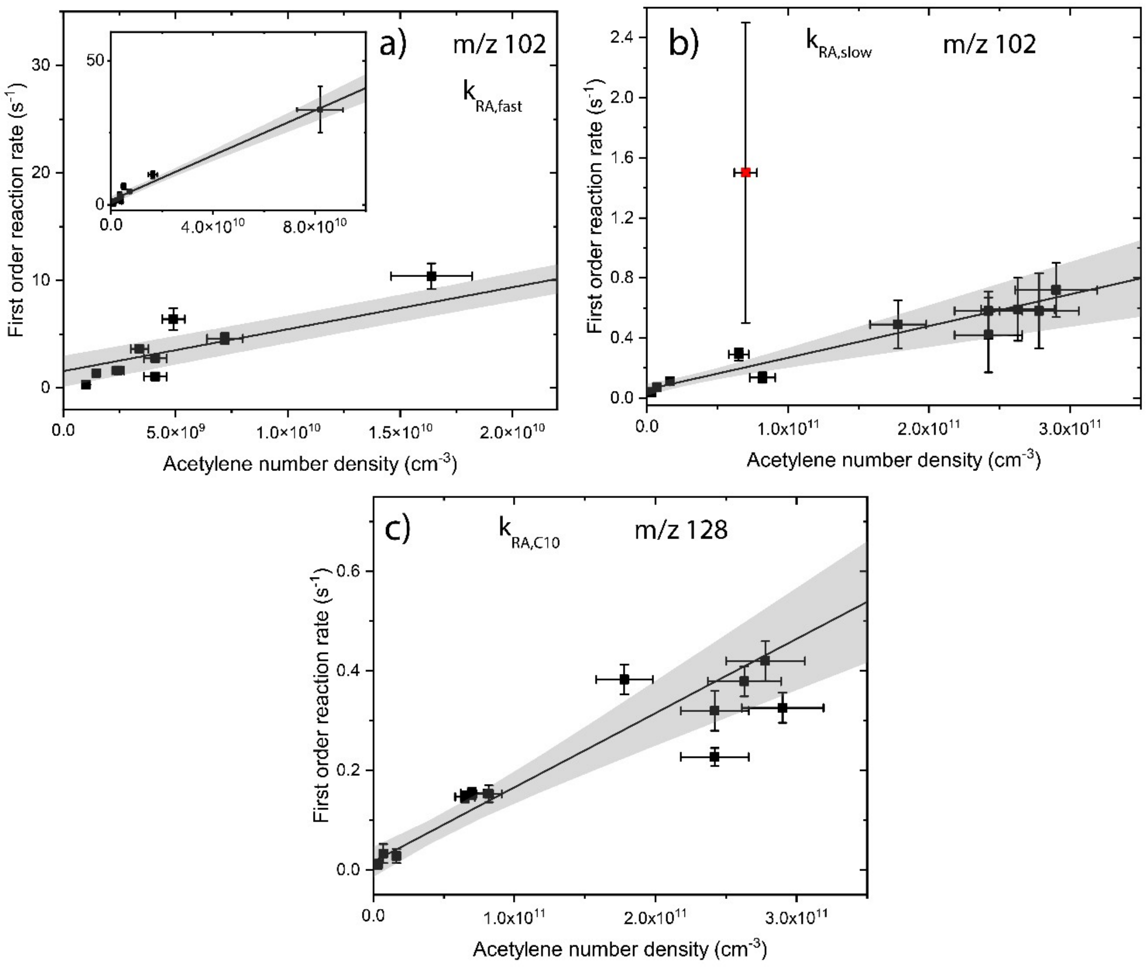

Supplementary Fig. 4: Second-order reaction rate coefficients obtained by a linear fit using the first-order rate coefficients from both the reduced and biexponential ODE models: (a) $k_{RA,fast}$ to m/z 102 (b) $k_{RA,slow}$ to m/z 102 and (c) $k_{RA,C10}$ to m/z 128. The error bars indicate the 1σ errors and the grey shaded bands define the 2σ uncertainty regions of the fits.

Supplementary Tab. 1: Comparison between the rate coefficients obtained from using the reduced ODE model, the biexponential ODE model and the fitted results of both models combined. Values of the rate coefficient are in $cm^3\ s^{-1}$.

Reaction	Label	Rate coefficient:		
		**Reduced ODE model**	**Biexponential ODE model**	**Both models**
$76^{\cdot+} \rightarrow 102^{\cdot+}$	$k_{RA,fast}$	$2.7(\pm0.7) \cdot 10^{-10}$	$3.8(\pm0.2) \cdot 10^{-10}$	$3.9(\pm0.2) \cdot 10^{-10}$
$76^{\cdot+} \rightarrow 102^{\cdot+}$	$k_{RA,slow}$	$1.3(\pm0.4) \cdot 10^{-12}$	$1.0(\pm0.7) \cdot 10^{-12}$	$2.1(\pm0.3) \cdot 10^{-12}$
$102^{\cdot+} \rightarrow 128^{\cdot+}$	$k_{RA,C10}$	$1.5(\pm0.2) \cdot 10^{-12}$	$2.1(\pm0.2) \cdot 10^{-12}$	$1.5(\pm0.2) \cdot 10^{-12}$

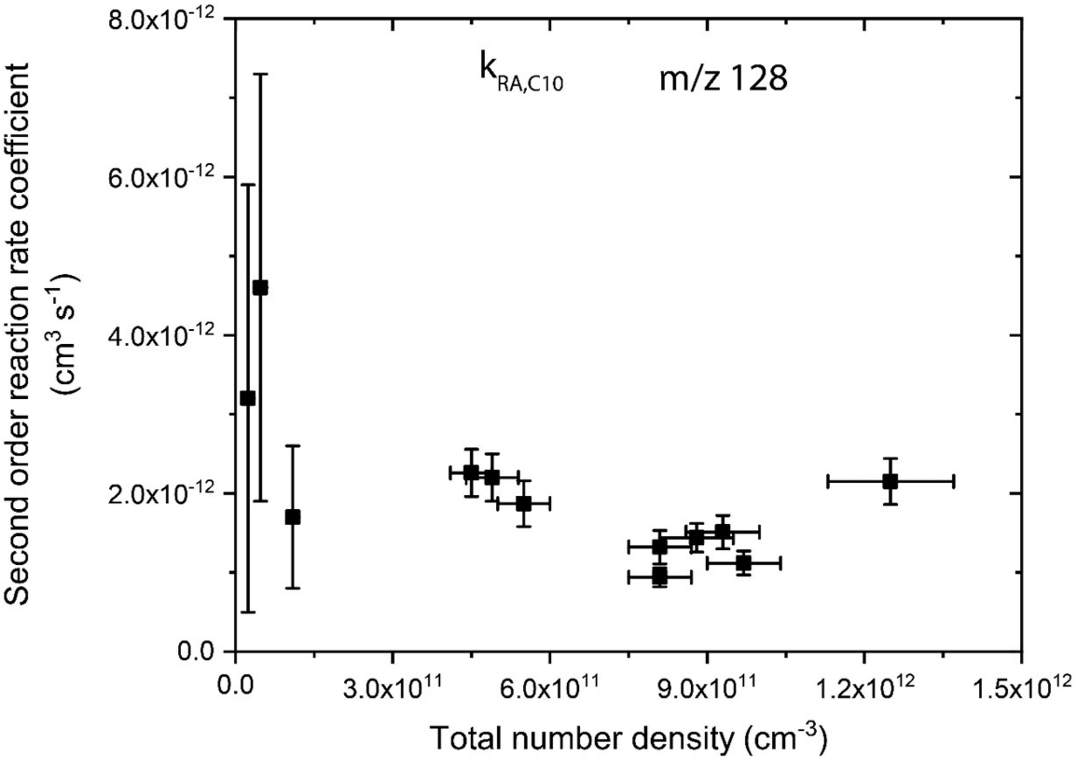

Supplementary Fig. 5: Second-order reaction rate coefficient of the reaction to m/z 128 plotted against the total number density of acetylene and helium. The error bars indicate the 1σ errors. The first points are less defined due to the lower acetylene number density and lower yield of m/z 128. Assuming no saturation, the effective bimolecular rate constant k_{EFF-BI} can be described according to formula $k_{EFF-BI} = k_{RA} + k_{TER}[B]$ with k_{RA} the radiative association rate constant, k_{TER} the termolecular collisional stabilization, and [B] the total number density. Across the number density range, no increase in the reaction rate coefficient is observed, which indicates that termolecular stabilization (k_{TER}) is negligible. The determined bimolecular rate coefficient is therefore dominated by a radiative association process.

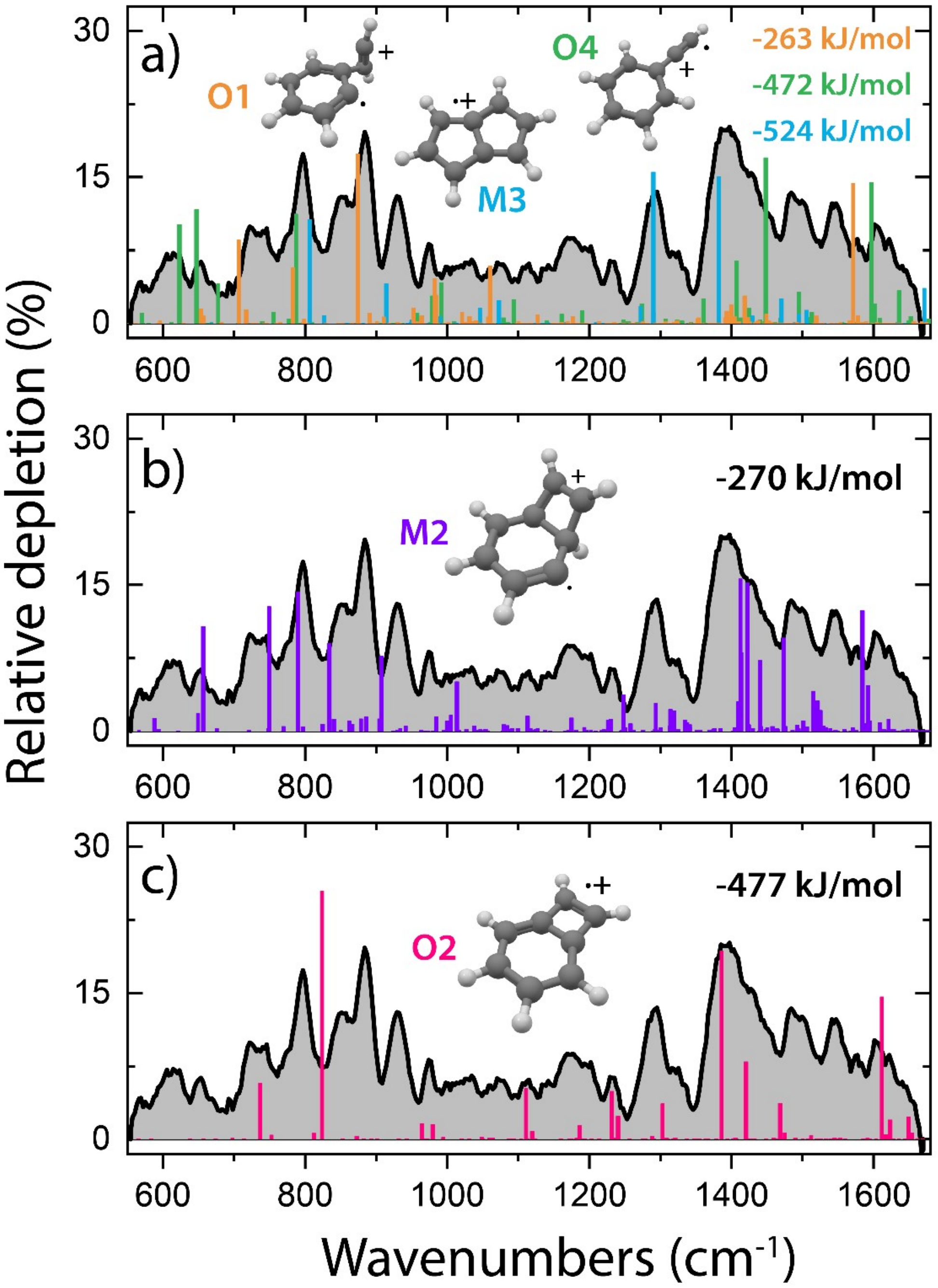

Supplementary Fig. 6: Experimental infrared fingerprint spectrum (grey) of the intermediate with m/z 102 with experimental conditions where the product with m/z 128 is not formed. Calculated vibrational modes are shown for (a) the assigned phenylacetylene$^{\bullet+}$ (**O4**, green), covalent acetylene ortho-benzyne$^{\bullet+}$ complex (**O1**, orange) and pentalene$^{\bullet+}$ (**M3**, blue), (b) covalent acetylene meta-benzyne$^{\bullet+}$ complex (**M2**, purple) and (c) benzocyclobutadiene$^{\bullet+}$ (**O2**, pink). The calculations have been performed at the anharmonic B3LYP-GD3/N07D level of theory. The zero-point vibrational energy corrected electronic energies of the molecules are shown relative to the energy of the entrance channel.

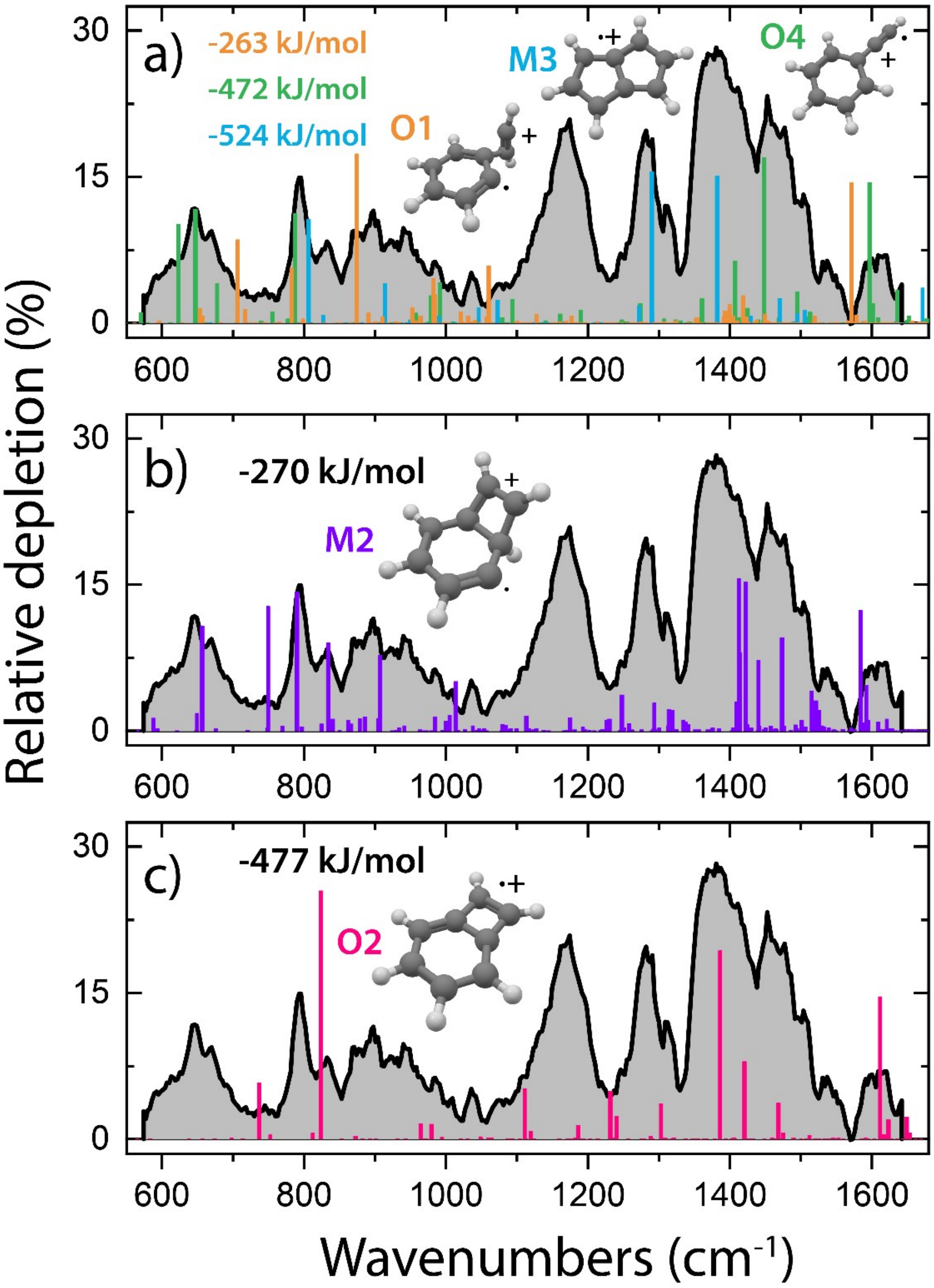

Supplementary Fig. 7: Experimental infrared fingerprint spectrum (grey) of the intermediate with m/z 102 with experimental conditions where the product with m/z 128 is formed. Calculated vibrational modes are shown for (a) the assigned phenylacetylene$^{•+}$ (**O4**, green), covalent acetylene ortho-benzyne$^{•+}$ complex (**O1**, orange) and pentalene$^{•+}$ (**M3**, blue), (b) covalent acetylene meta-benzyne$^{•+}$ complex (**M2**, purple) and (c) benzocyclobutadiene$^{•+}$ (**O2**, pink). The calculations have been performed at the anharmonic B3LYP-GD3/N07D level of theory. The zero-point vibrational energy corrected electronic energies of the molecules are shown relative to the energy of the entrance channel.

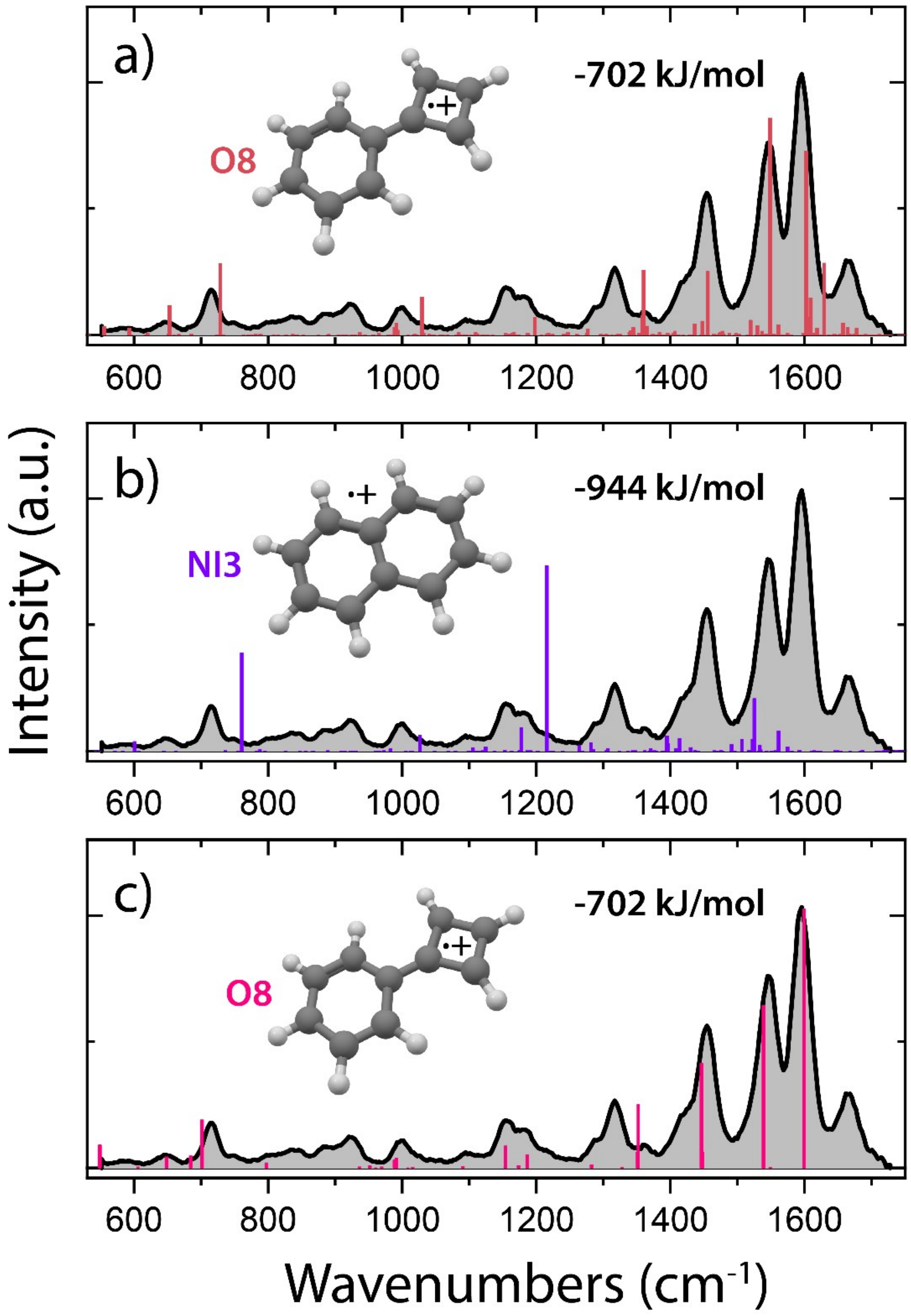

Supplementary Fig. 8: Experimental infrared fingerprint spectrum (grey) of the product with m/z 128. Calculated vibrational modes are shown for (a) the assigned phenylcyclobutadiene$^{\bullet+}$ (**O8**, red) and (b) naphthalene$^{\bullet+}$ (**NI3**, purple). The calculations have been performed at the anharmonic B3LYP-GD3/N07D level of theory. A comparison with calculated harmonic vibrational modes at the B3LYP/6-311++G(2d,p) level of theory of phenylcyclobutadiene$^{\bullet+}$ (**O8**, pink) is shown in panel c. The zero-point vibrational energy corrected electronic energies of the molecules are shown relative to the energy of the entrance channel.

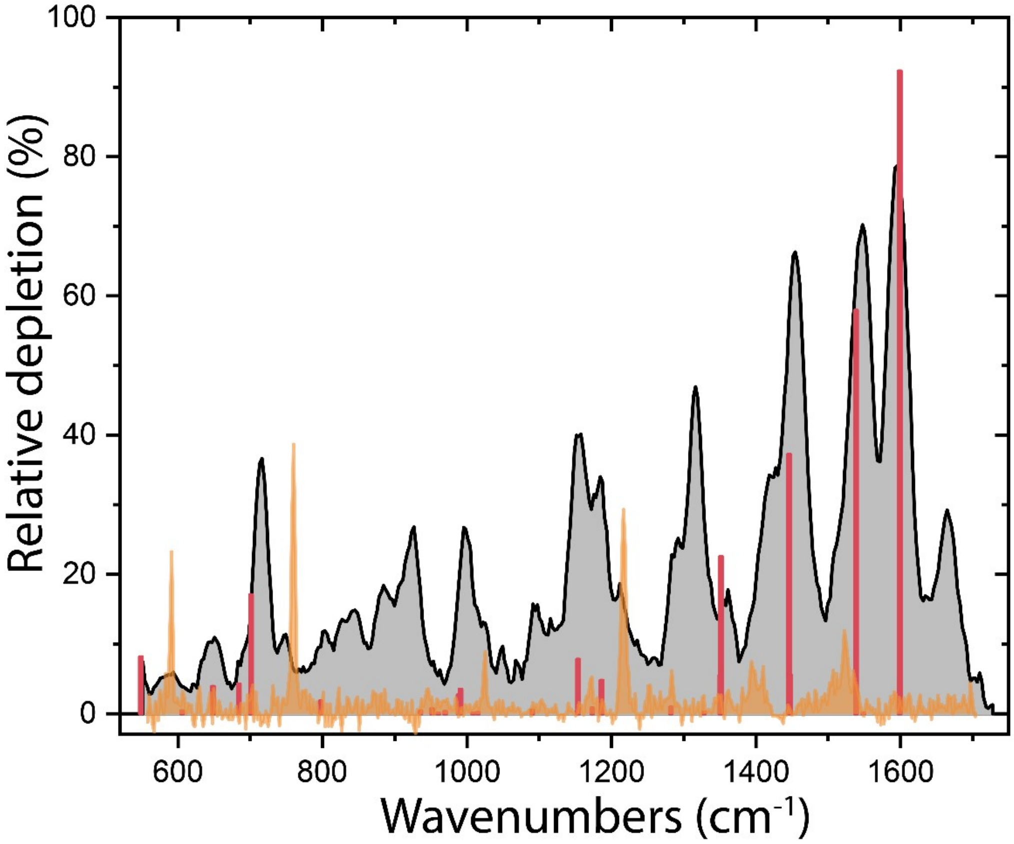

Supplementary Fig. 9: Comparison between the experimental infrared spectrum of the product with m/z 128 from the reaction of o-benzyne$^{\bullet+}$ (**O**)/m-benzyne$^{\bullet+}$ (**M**)/ethynyl-methylene-cyclopropene$^{\bullet+}$ (**E**) with acetylene and an experimental reference spectrum (orange) of naphthalene$^{\bullet+}$ (unpublished results). The experimental intensities are shown as relative depletion. Calculated harmonic vibrational modes at the B3LYP/6-311++G(2d,p) level of theory (red sticks) are shown.

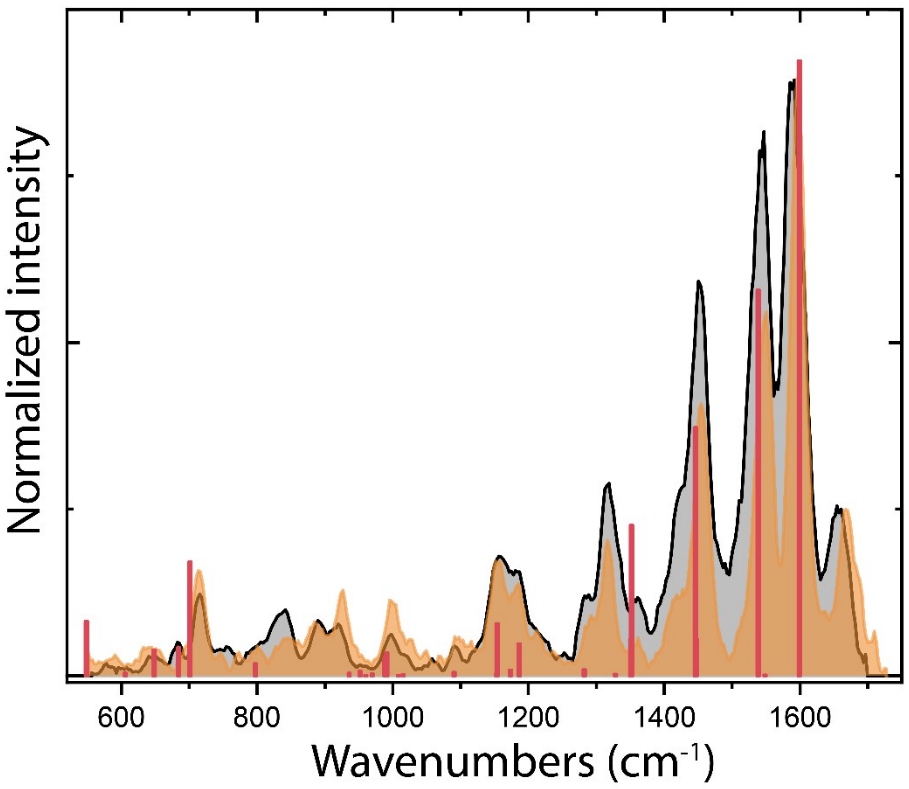

Supplementary Fig. 10: Comparison between the experimental infrared spectra of the m/z 128 product formed by the reaction of o-benzyne$^{•+}$ (**O**)/m-benzyne$^{•+}$ (**M**)/ethynyl-methylene-cyclopropene$^{•+}$ (**E**) with acetylene using both the direct source (orange) and storage ion source (black) to generate the m/z 76 reactant. Calculated harmonic vibrational modes at the B3LYP/6-311++G(2d,p) level of theory (red sticks) are shown.

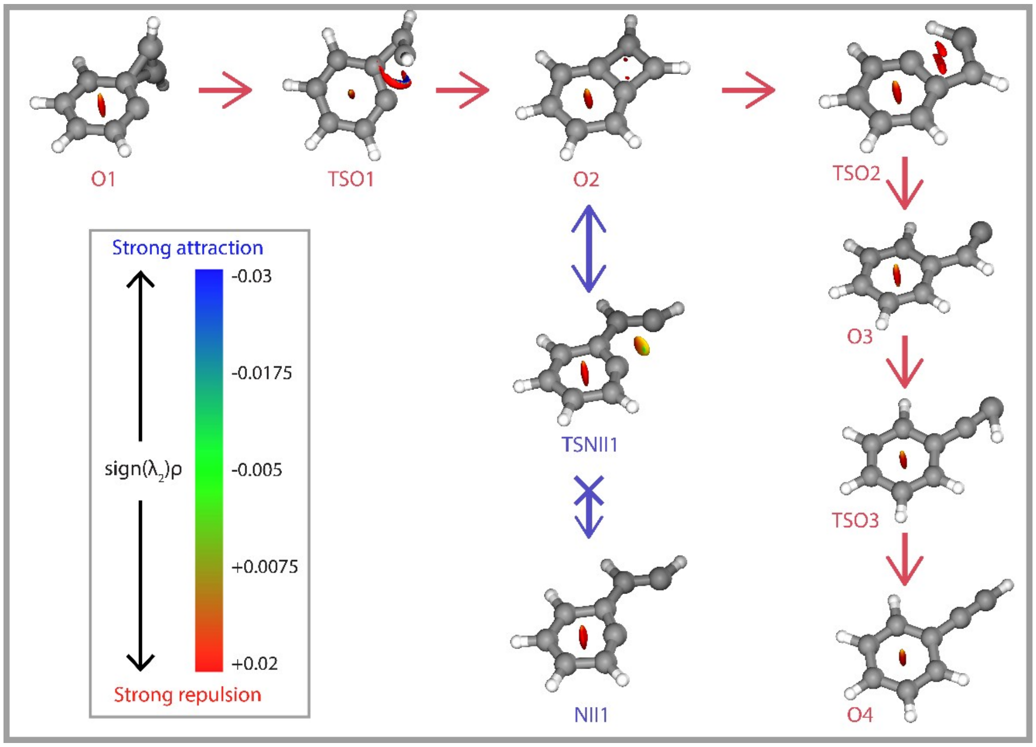

Supplementary Fig. 11: NCI plots of intermediates and transition states of the reaction pathways towards phenylacetylene$^{\bullet+}$ (**O4**, red arrows) and acetylene-o-benzyne$^{\bullet+}$ (**NII1**, blue arrows). The strengths of the NCIs are shown by the color spectrum ranging from red (strong repulsion), green (weak attraction) and blue (strong attraction).

Supplementary Tab. 2: Calculated harmonic oscillator model of aromaticity (HOMA)[1] values for relevant neutral and radical cationic molecules. Negative values correspond to anti-aromaticity, values around zero indicate non-aromaticity and values close to 1 indicate full aromaticity. The values for phenylcyclobutadiene are separated into the HOMA of the six-membered and four-membered ring, respectively.

Molecule	HOMA (neutral)	HOMA (radical cation)
benzene	0.969	0.629
naphthalene	0.817	0.883
pentalene	-0.377	0.765
phenylcyclobutadiene	0.930/-3.505	0.835/0.018

References

1 T. M. Krygowski, *J. Chem. Inf. Comput. Sci*, 1993, **33**, 70–78.
